# Sleep Duration, Dietary Inflammatory Potential, and Obesity in Relation to Colorectal Cancer Incidence in the Multiethnic Cohort

**DOI:** 10.3390/nu17030370

**Published:** 2025-01-21

**Authors:** Penias Tembo, Longgang Zhao, Loïc Le Marchand, Lynne R. Wilkens, Song-Yi Park, Christopher A. Haiman, Michael D. Wirth, James R. Hébert

**Affiliations:** 1 Department of Epidemiology and Biostatistics and Cancer Prevention and Control Program, University of South Carolina, Columbia, SC 29208, USA; ptembo@email.sc.edu (P.T.); wirthm@email.sc.edu (M.D.W.); 2 School of Nursing, Yale University, Orange, CT 06477, USA; longgang.zhao@yale.edu; 3 Population Sciences in the Pacific Program, University of Hawai’i Cancer Center, University of Hawai’i at Mānoa, Honolulu, HI 96822, USA; loic@cc.hawaii.edu (L.L.M.); lynne@cc.hawaii.edu (L.R.W.); spark@cc.hawaii.edu (S.-Y.P.); 4 Department of Population and Public Health Sciences, Keck School of Medicine, University of Southern California, Los Angeles, CA 90007, USA; christopher.haiman@med.usc.edu; 5 College of Nursing, University of South Carolina, Columbia, SC 29208, USA; 6 Department of Nutrition, Connecting Health Innovations, LLC, Columbia, SC 29201, USA

**Keywords:** sleep duration, energy-adjusted dietary inflammatory index (E-DII), obesity, colorectal cancer (CRC), multiethnic cohort (MEC)

## Abstract

Background/Objectives: Colorectal cancer (CRC) is a leading cause of cancer-related morbidity and mortality worldwide. Sleep duration, diet, and obesity have each been identified as modifiable risk factors linked to CRC. However, their joint effect on CRC incidence is underexplored. This study investigated the association between sleep duration and CRC incidence and explored the joint effects of sleep duration, a pro-inflammatory diet, and obesity on CRC incidence in the Multiethnic Cohort (MEC). Methods: This prospective cohort study analyzed 193,027 participants from Hawaii and California enrolled in the MEC between 1993 and 1996. Sleep duration was self-reported and categorized as short (≤6 h), normal (7–8 h), or long (≥9 h). Diet was self-reported via FFQ and inflammatory potential was assessed using the energy-adjusted Dietary Inflammatory Index (E-DII). CRC cases were identified via cancer registries. Cox proportional hazards models estimated the hazard ratios (HRs) for CRC risk. Results: After 23.8 years of follow-up, 5825 CRC cases were identified. A pro-inflammatory diet combined with suboptimal sleep increased CRC risk by 12% (short sleep duration, aHR: 1.12; 95% CI: 1.02–1.24) and 22% (long sleep duration, aHR: 1.22, 95% CI: 1.05–1.43). Furthermore, long sleep duration was associated with a 10% increase in CRC risk (aHR: 1.10; 95% CI: 1.01–1.22) compared with normal sleep, while short sleep showed no significant association overall. Obese individuals with short or long sleep had significantly higher CRC risk (short sleep aHR: 1.35; 95% CI: 1.21–1.51; long sleep aHR: 1.36; 95% CI: 1.14–1.64) compared with non-obese individuals with corresponding sleep durations. Conclusions: Long sleep duration and a combination of suboptimal sleep duration and a pro-inflammatory dietary pattern or obesity amplifies the risk.

## 1. Introduction

Colorectal cancer (CRC) ranks as the third most frequently diagnosed cancer globally, representing around 10% of all cancer cases, and is the second leading cause of cancer-related mortality worldwide [1]. The American Cancer Society estimates that in the United States, there will be approximately 153,000 new cases and about 53,000 deaths attributed to CRC in 2024 [2]. The distribution of CRC varies significantly among different subpopulations in the United States, with notable disparities in incidence and mortality rates across racial and ethnic groups. A previous study linking the Multiethnic Cohort (MEC) with the Surveillance, Epidemiology, and End Results (SEER) program revealed that Japanese-American men and women, as well as African-American women, had a higher risk of colorectal cancer compared with White individuals [3]. Consistent with these findings, other studies have reported that African Americans have the highest incidence and mortality rates of CRC compared with other major racial and ethnic groups in the country [4,5].

Advancements in screening and treatment modalities have significantly reduced overall CRC incidence and mortality, particularly among older populations [6]. However, sustaining and enhancing these preventive efforts requires a deeper understanding of lifestyle factors linked to chronic inflammation, given its central role in CRC development [7]. While non-modifiable risk factors such as age, sex, genetic predisposition, and family history remain important determinants of CRC risk, lifestyle factors like diet [8] and sleep [9] stand out as those with strong inflammatory underpinnings. Indeed, there exists a direct and bidirectional relationship between inflammatory cytokines and sleep [10,11].

Sleep is a known physiological necessity. Adults aged 18 to 60 years are advised to get approximately 7 to 8 h of sleep each night on a regular basis to achieve optimal health [12]. A study evaluating 15-year trends in self-reported sleep duration among adults revealed significant racial and ethnic disparities [13]. Such deviations have been associated with adverse outcomes. For example, a meta-analysis by Chen et al. found that insufficient sleep heightened cancer risk among Asians, while excessive sleep was linked to CRC [14]. Similarly, a meta-analysis by Lu et al. found that long sleep duration increased the risk of CRC [15]. An increased risk of other types of cancers has been linked to inadequate sleep duration. For example, a nine-year prospective cohort study in China reported a 27% higher cancer risk, especially for both digestive and respiratory cancers, among those sleeping fewer than six hours nightly [16]. The NIH-AARP Diet and Health Study found that men sleeping only 5–6 h per night faced a significantly higher risk of stomach cancer [17]. These findings highlight the critical role of consistent, optimal sleep duration in reducing cancer risk, though further research is needed to understand the mechanisms behind these associations.

Diet is another factor that is strongly associated with chronic inflammation. There is a consistent association between consumption of pro-inflammatory diets and an elevated risk of cancer [18,19]. Furthermore, pro-inflammatory dietary patterns are linked to obesity [20], which, in turn, is implicated in at least 13 different cancer types [21]. Lifestyle factors that are closely related to inflammation may interact in ways that amplify CRC risk. These interactions are particularly relevant given variations in dietary habits, sleep patterns, and obesity levels across different ethnic groups [13,22,23]. This study examined the association between sleep duration and CRC incidence within the MEC. It also examined the joint effect of sleep duration and diet-associated inflammation, as well as sleep duration and obesity, on CRC incidence, recognizing the interconnected role of these factors in modulating inflammation and influencing cancer development.

## 2. Materials and Methods

### 2.1. Study Population

The composition and recruitment criteria of the MEC has been described previously [24]. This prospective cohort study included men and women 45–75 years of age, primarily from five racial and ethnic groups: White/European American, Japanese American, Native Hawaiian, Black/African American, and Latino. The cohort at baseline consisted of 215,251 participants, who were recruited between 1993 and 1996. Most African Americans and Latinos in the cohort were recruited from California, while the majority of Japanese Americans, European Americans, and Native Hawaiians were recruited from Hawaii [24]. Driver’s licenses, voter registration lists, and Medicare documents were used to identify potential participants. An initial survey was administered via a 26-page questionnaire, which included demographic, anthropometric, dietary intake, and other lifestyle factors. The MEC protocol was approved by the institutional review boards at the University of Hawaii and the University of Southern California.

### 2.2. Inclusion and Exclusion Criteria

**Figure 1:**
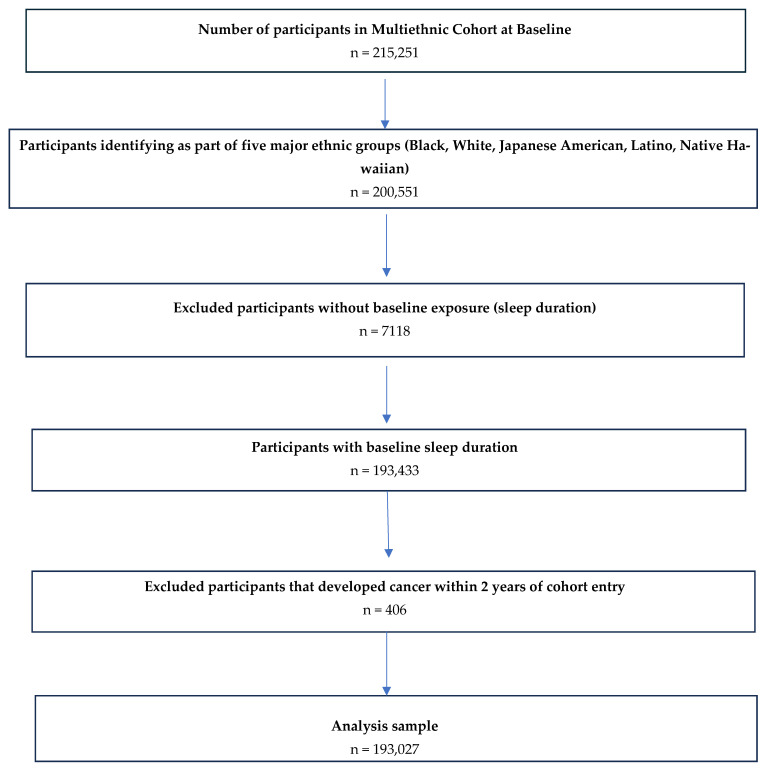
Inclusion criteria utilized for final study population to examine the association between sleep duration and colorectal cancer incidence in the MEC.

Of the 215,251 participants recruited at baseline, 200,551 were eligible for this study based on their identification as members of one of the five major racial and ethnic groups. From this group, we excluded participants who did not provide data on their baseline sleep duration (*n* = 7118) or who developed CRC within the first 2 years of cohort entry (*n* = 406). After applying our inclusion and exclusion criteria, we had an analytical sample of 193,027 participants (Figure 1).

### 2.3. Exposures

This study utilized three key exposures. First, self-reported sleep duration was analyzed as an independent exposure. Next, a combined exposure was created by jointly considering sleep duration and obesity status. Similarly, a third combined exposure was created by jointly considering sleep duration and diet quality as measured by the energy-adjusted Dietary Inflammatory Index (E-DII^TM^).

Self-reported sleep duration was obtained from the baseline questionnaire. Each participant was asked the following question: “On the average, during the last year, how many hours in a day did you sleep (include naps)?” Participants were provided the following responses: “5 h or less”, “6 h”, “7 h”, “8 h”, “9 h”, and “10 h or more”. All data were reported in integer hours. In accordance with the 2015 consensus statement issued by the American Academy of Sleep Medicine, we categorized 7 to 8 h as adequate (normal) sleep [25]. Furthermore, we categorized less than or equal to 6 h of sleep as short sleep duration, while 9 h or more was categorized as long sleep duration.

The inflammatory potential of the diet was calculated from self-reports of dietary intake based on the DII and the E-DII scoring algorithms, which were developed to quantify the inflammatory potential of individuals’ diets on a scale from maximally anti-inflammatory (most negative score) to maximally pro-inflammatory (most positive score). The development of the DII has been described in detail elsewhere [26]. Briefly, the DII scoring algorithm was based on a careful review of the literature through which 1943 articles identified 45 food parameters (i.e., macronutrients, including specific categories of fatty acids, carbohydrates, and proteins; micronutrients, including vitamins and minerals; flavonoids; and whole food items, including herbs and spices) as having sufficiently robust literature in relation to six inflammatory biomarkers—i.e., interleukin (IL)-1b, -4, -6, -10; tumor-necrosis factor-alpha (TNFα); and C-reactive protein (CRP). Self-report values for 28 of these food parameters were available from the validated quantitative food frequency questionnaire developed for MEC [24,27,28]. These were translated into z-scores using a global comparative database consisting of data from 11 countries by subtracting from the individual’s self-report value the mean of the global database and then dividing by the standard deviation. These scores were then converted to proportions (i.e., with values ranging from 0 to 1) and centered on 0 by doubling each and subtracting 1. These centered proportions were then multiplied by their respective coefficients (overall food parameter–specific inflammatory effect scores) to obtain DII scores for each food parameter. These were summed to obtain the overall DII score. E-DII scores were calculated using the density approach by calculating DII per 1000 kcal consumption. This employed the same procedure for scoring but relied on an energy-adjusted global comparison database [19,29]. These DII and E-DII scores have a potential range from approximately −9 to +8, i.e., from minimally to maximally pro-inflammatory, respectively. The DII and E-DII are scored similarly and scaled identically, so the scores are comparable across studies [29].

For this study, the following 28 of the 45 food parameters were used to calculate an individual’s overall E-DII/DII score: carbohydrate; protein; total fat; saturated, monounsaturated, and polyunsaturated fats; ω-3 and ω-6 FAs; alcohol; fiber; cholesterol; vitamins A, B-6, B-12, C, D, and E; thiamin; riboflavin; niacin; iron; magnesium; zinc; selenium; folate; β-carotene; isoflavones; and caffeine. Food components not included were eugenol, garlic, ginger, onion, trans fat, turmeric, green tea, black tea, falan-3-ol, flavones, flavonols, flavanones, anthocyanins, pepper, thyme, oregano, and rosemary. The decision to use the E-DII was based on overall model explanatory ability and is consistent with our previous work on CRC in the MEC [19].

Body mass index (BMI) was obtained by dividing the participants’ measured weight in kilograms by their height in square meters (BMI= weight (kg)/height (m^2^)), based on self-reported weight and height. We then classified them as either obese (BMI ≥ 30 kg/m^2^) or non-obese (BMI < 30 kg/m^2^).

### 2.4. Outcomes

The outcome of interest was invasive CRC as reported to the National Cancer Centers Surveillance, Epidemiology, and End Results (SEER) program. The date of CRC diagnosis was taken as the event time for CRC cases, which were identified from two years after participants’ entry into the cohort up to December 2019. This approach excluded individuals who developed CRC within the first two years of cohort entry to minimize potential reverse causation. Follow-up time for non-cases was up to time of death, withdrawal from the study, or no evidence of CRC by censoring time (December 2019). Mortality from cancer and other causes was determined through linkages to death certificate records in Hawaii and California. The National Death Index was also used periodically to identify deaths among cohort members who had moved to other parts of the United States [24].

### 2.5. Covariates

The following covariates were included based on their associations with CRC from the literature [30]: age, sex, marital status, ethnicity, education, physical activity (measured as metabolic equivalent of task (MET) for activities performed over a typical 24 h period, relative to a MET value of 1 for sitting), smoking status, family history of colon cancer, previous disease diagnosis (heart disease, stroke, and diabetes) and hormone use (women only).

### 2.6. Statistical Analysis

Frequencies and percentages for baseline characteristics by sleep duration were computed. Cox proportional hazards models were fitted to estimate hazard ratios (HRs) and 95% confidence intervals (CIs) for sleep duration in relation to CRC incidence. Missing data for each variable were categorized as a separate group, ensuring that participants with missing information were retained in the analysis and their contribution to the risk estimates was accounted for. Time (in years) elapsed since study entry was used as the time metric. Kaplan–Meier survival curves were inspected for visual confirmation of proportional hazards across sleep duration groups. Sleep duration of 7 to 8 h was used as the reference group. Analyses were also conducted separately for each racial and ethnic group. A joint analysis was conducted to estimate the effect of sleep duration and obesity status on risk of CRC incidence and that of sleep duration and E-DII score on risk of CRC incidence. We created a dichotomous variable for obesity. Both the median E-DII score and an E-DII score of zero were used for separate joint analyses. The median E-DII score represents the midpoint of dietary inflammatory potential in the study population, providing a population-specific benchmark for categorization. An E-DII score of 0 represents a neutral inflammatory potential, categorizing a dietary pattern as pro-inflammatory (≥0) or anti-inflammatory (<0). To test for interaction, we included a product term of sleep duration and obesity status or sleep duration and E-DII score in the Cox proportional hazards models. The statistical significance of the interaction term was assessed based on the Wald test. A *p*-value < 0.05 was considered evidence of interaction. All analyses were performed using Stata^®^ version 16.

## 3. Results

A total of 193,027 participants were included in this study. Participant characteristics were compared based on sleep duration: 34.3% (*n* = 66,194) reported sleeping ≤6 h, 56.5% (*n* = 108,986) reported sleeping 7–8 h, and 9.2% (*n* = 17,847) reported sleeping ≥9 h (Table 1). Ethnic distribution varied by sleep duration, with Japanese Americans comprising the largest proportion among short sleepers (31.5%) and White/European Americans the largest proportion among long sleepers (28.1%). The mean age at cohort entry was 59.8 ± 8.8 years, and the mean age at CRC diagnosis was 74.5 ± 9.0 years. During a total of 3,875,479 person-years of follow-up (median follow-up time of 23.8 years), 5825 (3.0%) incident cases of invasive CRC were identified. Among the CRC cases, 2038 (35%) occurred in short sleepers, 3215 (55%) in normal sleepers, and 572 (10%) in long sleepers. The mean BMI was 26.6 ± 5.1 kg/m^2^, with approximately 39% of the participants having a BMI in the normal range (18.5–24.9 kg/m^2^). The mean E-DII score was −1.4 ± 2.0.

The association between short sleep duration (≤6 h) and CRC incidence was non-significant in the fully adjusted model (model 2, aHR: 1.04; 95% CI: 0.98–1.10 compared with 7–8 h). Long sleep duration (≥9 h) was associated with a 10% increased risk of CRC incidence (model 2, aHR: 1.10; 95% CI: 1.01–1.22) (Table 2). When stratified by ethnicity, both short and long sleep durations were not significantly associated with CRC incidence, except among Latinos, in whom long sleep duration was associated with a 22% risk of CRC incidence (aHR: 1.22; 95% CI: 1.01–1.48). There were no statistically significant differences in CRC incidence risk by age, sex, and E-DII quartile. However, a 17% increased risk (aHR: 1.17; 95% CI: 1.02–1.34) was observed among individuals with short sleep duration who were obese in comparison with those who had a normal BMI (Supplementary Table S1).

Among non-obese individuals, sleep duration was not associated with a risk of CRC incidence, with aHRs of 0.99 (95% CI: 0.93–1.06) for short sleep and 1.09 (95% CI: 0.97–1.22) for long sleep compared with normal sleep (Table 3). However, among obese participants, short sleep duration was associated with a 35% higher risk of CRC incidence (aHR: 1.35; 95% CI: 1.21–1.51), and long sleep duration was similarly associated with an increased risk (aHR: 1.36; 95% CI: 1.14–1.64) compared with non-obese individuals with normal sleep duration. Normal sleep duration in obese participants also showed a modestly increased CRC risk (aHR: 1.16; 95% CI: 1.06–1.29) compared with non-obese. Both short and long sleep durations, when combined with an E-DII score ≥ 0, were associated with an increased risk of CRC incidence. Short sleep duration combined with an E-DII score ≥ 0 was associated with a 12% increased risk of CRC incidence (aHR: 1.12; 95% CI: 1.02–1.24), while long sleep duration was associated with 22% increased risk of CRC incidence (aHR: 1.22; 95% CI: 1.05–1.43), compared with normal sleep duration combined with an E-DII score < 0.

## 4. Discussion

This study examined the association between sleep duration, diet-associated inflammation, obesity, and CRC incidence using a large, ethnically diverse cohort. We found that long sleep duration (≥9 h) compared with normal sleep duration (7–8 h) was associated with an increased risk of CRC incidence after adjusting for confounders. Additionally, the joint effect of suboptimal sleep duration with obesity and diet-associated inflammation revealed a statistically significant amplification in the risk of CRC incidence.

There is consistent evidence from both cohort and case-control studies that longer sleep duration is associated with an increased risk of CRC. For example, in the Women’s Health Initiative (WHI), long sleep duration was associated with a 47% increase in the risk of CRC in postmenopausal women [31]. Furthermore, in the Health Professionals Follow-up Study (HPFS), longer sleep duration was associated with a 35% increased risk of developing CRC [32]. The risk of CRC reported in both the WHI and HPFS studies was higher than the 10% increased risk observed in our study. However, our findings are consistent with a recent meta-analysis of seven cohort studies, which reported an 11% increased risk of CRC associated with longer sleep duration [33].

The mechanism through which long sleep duration is associated with CRC incidence is not well elucidated, but several plausible pathways have been proposed. For instance, disruption of circadian rhythms could impair DNA repair processes and promote tumor genesis [34]. A long sleep duration has been associated with high levels of pro-inflammatory cytokines, which create a pro-tumorigenic environment [35]. Moreover, prolonged sleep could lead to insulin resistance, which has been implicated in CRC development [36]. Additionally, long sleep duration is also associated with depression, which may contribute to residual confounding and could be a relevant factor to consider in future research [37].

In contrast, short sleep duration as a single exposure was not associated with CRC incidence, but its joint effect with obesity revealed a 35% increased risk. Studies have shown that short sleep duration alters appetite-regulating hormones, reducing leptin and increasing ghrelin levels, which disrupts appetite and subsequently may lead to obesity [38,39]. On the other hand, obesity, which is a well-established risk factor for CRC, may promote tumorigenesis through chronic inflammation, insulin resistance, and altered adipokine regulation [40]. When coupled with short sleep duration, these mechanisms may be exacerbated, amplifying the risk of CRC. We observed a similar joint effect among individuals classified as long sleepers who were obese. Limited studies have explored this joint effect. These findings suggest that obesity may act as a modifier, amplifying the adverse effects of both insufficient and excessive sleep durations on CRC incidence.

A previous study of the MEC by Harmon et al. that explored the association between the E-DII and CRC showed that pro-inflammatory diets were associated with an increased risk of CRC incidence [19]. Consumption of pro-inflammatory, energy-dense, often high-fat diets modulate the microbiota and induce alterations in intestinal barrier function that are associated with an increase in low-grade inflammation and insulin resistance [41,42,43,44]. Gut microbiota likely plays a central role in the connection between metaflammation, a chronic low-grade inflammation in metabolically active organs, and CRC [45,46,47]. When jointly considered with suboptimal sleep duration, the combined effects of pro-inflammatory diets and both short and long sleep durations appear to exacerbate the risk. This is likely mediated through mechanisms such as gut microbiota dysbiosis, heightened systemic inflammation, disruption of circadian rhythms, and metabolic dysfunction, which together promote a pro-inflammatory and pro-carcinogenic environment [48,49,50]. These findings emphasize the importance of addressing both dietary quality and sleep health as interconnected factors in CRC prevention.

Our study had several strengths that merit discussion. First, the use of a prospective design with a long follow-up time for a large, ethnically diverse population increases the generalizability of our findings. Second, sleep duration was obtained prior to diagnosis of CRC, which reduced the likelihood of recall bias. Third, cancer diagnoses were obtained from verified registries, which reduced the likelihood of diagnostic misclassification. Fourth, the large number of covariates allowed the adjustment for many known risk factors associated with CRC. Despite these strengths, our study was limited by the self-reported nature of the sleep duration and other covariates (e.g., diet, BMI) and the absence of other essential sleep-related covariates such as sleep quality, snoring, daytime drowsiness, nightshift work, and depression. Previous studies have shown that individuals tend to overreport their self-reported sleep duration [51]. Furthermore, the exposure assessment occurred over two decades ago, and lifestyle patterns may have shifted since then. This temporal gap represents a limitation to our findings. However, given the inherent difficulty in altering deeply ingrained habits, our findings remain relevant today and provide important details on the long-term associations that are likely to persist over time. Additionally, our study was restricted to individuals at least 45 years of age. Therefore, our results are not generalizable to younger populations. Future studies should aim to understand how sleep duration affects cancer risk in populations under 45 years of age. This is especially critical, considering that early-onset CRC has been increasing at an alarming rate over the last few decades [52]. Efforts are currently underway to understand this phenomenon. For example, the Metabolic Dysregulation and Cancer Risk Program (MeDOC), a trans–National Cancer Institute research initiative, is working to advance our understanding of the underlying mechanisms that connect obesity, metabolic dysregulation, and increased cancer risk [53].

Our study highlights the important roles of sleep duration, dietary inflammation, and obesity as risk factors for CRC. Given the global rise in obesity, the widespread consumption of pro-inflammatory diets, and the high prevalence of both short and long sleep durations, these findings emphasize the urgent need for targeted public health strategies to address these interconnected risks [54,55]. Public health initiatives should focus on promoting optimal sleep duration while simultaneously advocating for the consumption of anti-inflammatory diets and supporting strategies to achieve and maintain a healthy weight, integrating these efforts into holistic approaches to reduce CRC risk.

## 5. Conclusions

We found that long sleep duration (≥9 h) was associated with a 10% increased risk of CRC incidence compared with participants with normal sleep duration (7–8 h). In a joint analysis, short sleep duration (≤6 h) and obesity were associated with a 35% increased risk of CRC incidence, while long sleep duration (≥9 h) and obesity were associated with a 36% increased risk, compared with those with normal sleep duration and no obesity. Furthermore, a combination of habitual short or long sleep duration and the consumption of a pro-inflammatory diet increased the risk of CRC incidence by 12% and 22%, respectively, compared with those with normal sleep duration and an anti-inflammatory diet.

## Figures and Tables

**Table 1:** Baseline characteristics and colorectal cancer incidence by sleep duration in the MEC (1993–2019).

Characteristic	Sleep Duration			Total
	≤6 h 66,194 (34.3%)	7–8 h 108,986 (56.5%)	≥9 h 17,847 (9.2%)	*n* = 193,027
Number of CRC Cases	2038	3215	572	5825
Geographic Location, *n* (%)				
Hawaii	30,961 (46.8)	54,122 (49.7)	7482 (41.9)	92,565 (47.9)
California	35,233 (53.2)	54,864 (50.3)	10,365 (58.1)	100,462 (52.1)
Age at Cohort Entry (y), *n* (%)				
45–54	21,960 (33.2)	34,884 (32.0)	4759 (26.7)	61,603 (31.9)
55–64	21,970 (33.2)	36,627 (33.6)	5694 (31.9)	64,219 (33.3)
≥65	22,264 (33.6)	37,475 (34.4)	7394 (41.4)	67,133 (34.8)
Sex of Participant, *n* (%)				
Male	28,885 (43.6)	50,117 (46.0)	8511 (47.7)	87,513 (45.3)
Female	37,309 (56.4)	58,869 (54.0)	9336 (52.3)	105,514 (54.7)
Ethnicity, *n* (%)				
Black/African American	13,290 (20.1)	15,074 (13.8)	3343 (19.2)	31,798 (16.5)
Native Hawaiian	5773 (8.7)	6835 (6.3)	1264 (7.1)	13,872 (7.2)
Japanese American	20,847 (31.5)	30,771 (28.2)	3222 (18.1)	54,840 (28.4)
Latino	14,528 (22.0)	25,096 (23.0)	4915 (27.5)	44,539 (23.1)
White/European American	11,756 (17.7)	31,210 (28.7)	5012 (28.1)	47,978 (24.8)
Education Level, *n* (%)				
Less than high school	11,797 (17.8)	17,819 (16.4)	4186 (23.5)	33,802 (17.5)
High school/vocational school	23,225 (35.1)	35,918 (33.0)	6311 (35.4)	65,454 (33.9)
College level	22,776 (34.4)	39,181 (36.0)	5421 (30.4)	67,378 (34.9)
Graduate/professional	7624 (11.5)	14,998 (13.8)	1686 (9.5)	24,308 (12.6)
Missing data	772 (1.2)	1070 (0.8)	243 (1.2)	2085 (1.1)
Marital Status, *n* (%)				
Never married	4666 (7.1)	7044 (6.5)	1203 (6.7)	12,913 (6.7)
Married	41,089 (62.1)	74,768 (68.6)	11,777 (66.0	127,634 (66.1)
Divorced/separated/widowed	19,876 (30.0)	26,340 (24.2)	4703 (26.4)	50,919 (26.4)
Missing data	563 (0.8)	834 (0.7)	164 (0.9)	1561 (0.8)
Body Mass Index (kg/m^2^), *n* (%)				
Underweight (<18.5)	1207 (1.8)	1765 (1.6)	312 (1.8)	3284 (1.7)
Normal weight (18.5 to 24.9)	24,392 (36.8)	45,073 (41.4)	6216 (34.8)	75,681 (39.2)
Overweight (25 to 29.9)	25,460 (38.5)	41,533 (38.1)	6779 (38.0)	73,772 (38.2)
Obese (≥30)	14,389 (21.7)	19,739 (18.1)	4320 (24.2)	38,448 (19.9)
Missing data	746 (1.2)	876 (0.8)	220 (1.2)	1842 (1.0)
E-DII Quartiles, *n* (%)				
Quartile 1 (−6.4 to −2.9)	16,443 (24.8)	29,223 (26.8)	3954 (22.1)	49,620 (25.7)
Quartile 2 (−2.8 to −1.5)	15,404 (23.3)	26,645 (24.5)	4026 (22.6)	46,075 (23.9)
Quartile 3 (−1.4 to 0.1)	15,354 (23.2)	25,355 (23.3)	4335 (24.3)	45,044 (23.3)
Quartile 4 (0.2 to 5)	16,059 (24.3)	24,202 (22.2)	4722 (26.5)	44,983 (23.3)
Missing data	2934 (4.4)	3561 (3.2)	810 (4.5)	7305 (3.8)
Smoking Status, *n* (%)				
Never	29,358 (44.4)	47,640 (43.7)	6801 (38.1)	83,799 (43.4)
Previous smoker	24,872 (37.6)	43,307 (39.7)	7462 (41.8)	75,641 (39.2)
Current smoker	10,887 (16.5)	16,619 (15.3)	3292 (18.5)	30,798 (16.0)
Missing data	1077 (1.5)	1420 (1.3)	292 (1.6)	2789 (1.4)
Estrogen use, *n* (%)				
Never	19,685 (25.7)	30,173 (27.7)	4914 (27.5)	54,772 (28.4)
Previous user	6882 (10.4)	9913 (9.1)	1695 (9.5)	18,490 (9.6)
Current user	9625 (14.5)	17,346 (15.9)	2451 (13.7)	29,442 (15.2)
Missing/not applicable (male)	30,002 (45.4)	51,554 (47.3)	8787 (49.3)	90,343 (46.8)
Progesterone use, *n* (%)				
Never	15,716 (23.7)	23,664 (21.7)	3953 (22.2)	43,333 (22.5)
Previous user	3368 (5.1)	5495 (5.0)	847 (4.8)	9710 (5.0)
Current user	3956 (6.0)	7770 (7.1)	1075 (6.0)	12,801 (6.6)
Missing/not applicable (male)	43,154 (65.2)	72,057 (66.2)	11,972 (67.0)	127,183 (65.9)
Dietary Supplement use, *n* (%)				
No	23,869 (36.1)	38,573 (35.4)	6990 (39.2)	69,432 (36.0)
Yes	42,325 (63.9)	70,413 (64.6)	10,857 (60.8)	123,595 (64.0)
Family History of Colon Cancer, *n* (%)				
No	51,887 (78.4)	87,493 (80.3)	14,010 (78.5)	153,390 (79.5)
Yes	5344 (8.1)	8543 (7.8)	1301 (7.3)	15,188 (7.9)
Missing	8963 (13.5)	12,950 (11.9)	2536 (14.2)	24,449 (12.6)
* Previous Chronic Condition, *n* (%)				
No	52,749 (76.7)	89,969 (82.6)	13,207 (74.0)	155,925 (80.8)
Yes	13,445 (20.3)	19,017 (17.4)	4640 (26.0)	37,102 (19.2)
Continuous Variables				
Age at Cohort Entry (years) (Mean ± SD)	59.6 ± 8.8	59.7 ± 8.8	61.2 ± 8.8	59.8 ± 8.8
Age at CRC Diagnosis (years) (Mean ± SD)	74.1 ± 9.1	74.6 ± 8.9	74.8 ± 8.8	74.5 ± 9.0
Body Mass Index (kg/m^2^) (Mean ± SD)	26.9 ± 5.3	26.3 ± 4.9	27.2 ± 5.5	26.6 ± 5.1
** Physical Activity (METs) (Mean ± SD)	1.7 ± 0.3	1.6 ± 0.3	1.5 ± 0.3	1.6 ± 0.3
E-DII Score (Mean ± SD)	−1.3 ± 2.0	−1.4 ± 1.9	−1.2 ± 2.0	−1.4 ± 2.0
Pack Years of Smoking, (Mean ± SD)	9.7 ± 14.6	10.1 ± 14.9	12.4 ± 16.6	10.4 ± 16.6
Alcohol Consumption (g/day), (Mean ± SD)	8.1 ± 25.1	9.2 ± 24.3	11.5 ± 30.2	9.0 ± 25.2

* Previous chronic conditions included either heart disease, stroke, or diabetes. ** Physical activity (measured as metabolic equivalent of tasks (METs) for activities performed over a typical 24 h period, relative to a MET value of 1 for sitting).

**Table 2:** Association between sleep duration and CRC incidence in the MEC (1993–2019).

	Sleep Duration		
	≤6 h	7–8 h	≥9 h
	HR (95% CI)	HR (95% CI)	HR (95% CI)
All Participants			
Model 1 (Crude)	1.08 (1.02,1.14)	1.00 (Reference)	1.15 (1.05,1.25)
Model 2 (Adjusted)	1.04 (0.98,1.10)	1.00 (Reference)	1.10 (1.01,1.22)
Black/African American			
Model 1 (Crude)	1.06 (0.94,1.20)	1.00 (Reference)	0.96 (0.78,1.19)
Model 2 (Adjusted)	1.02 (0.89,1.18)	1.00 (Reference)	0.87 (0.69,1.10)
Native Hawaiian			
Model 1 (Crude)	1.06 (0.96,1.16)	1.00 (Reference)	1.21 (1.01,1.45)
Model 2 (Adjusted)	1.15 (0.91,1.44)	1.00 (Reference)	0.99 (0.66,1.48)
Japanese American			
Model 1 (Crude)	0.96 (0.85,1.09)	1.00 (Reference)	1.25 (1.05,1.48)
Model 2 (Adjusted)	1.04 (0.94,1.15)	1.00 (Reference)	1.19 (0.99,1.44)
Latino			
Model 1 (Crude)	0.96 (0.85,1.09)	1.00 (Reference)	1.25 (1.05,1.49)
Model 2 (Adjusted)	1.02 (0.88,1.18)	1.00 (Reference)	1.22 (1.01,1.48)
White/European American			
Model 1 (Crude)	1.05 (0.91,1.20)	1.00 (Reference)	1.19 (0.99,1.42)
Model 2 (Adjusted)	0.99 (0.86,1.15)	1.00 (Reference)	1.13 (0.93,1.37)

Model 1: Age and sex-adjusted model. Model 2: Model 1 + ethnicity, education, marital status, BMI, E-DII Score, smoking status, pack year history, alcohol consumption, physical activity, hormone therapy (estrogen and progesterone), diet supplement use, family history of colon cancer, and previous chronic condition (heart disease, stroke, diabetes).

**Table 3:** Joint effects of sleep duration/obesity and sleep duration/E-DII score on CRC incidence in the MEC (1993–2019).

	Sleep Duration, aHR (95% CI)			
	Short (≤6 h)	Normal/Adequate (7–8 h)	Long (≥9 h)	*p* Interaction
Obesity				0.08
BMI < 30 kg/m^2^	0.99 (0.93,1.06)	1.00 (Reference)	1.09 (0.97,1.22)	
BMI ≥ 30 kg/m^2^	1.35 (1.21,1.51)	1.16 (1.06,1.29)	1.36 (1.14,1.64)	
E-DII Score				0.69
<−1.5 (median)	1.04 (0.96,1.13)	1.00 (Reference)	1.08 (0.94,1.25)	
≥−1.5 (median)	1.05 (0.97,1.14)	1.04 (0.97,1.12)	1.19 (1.05,1.34)	
E-DII Score				0.82
<0	1.01 (0.95,1.08)	1.00 (Reference)	1.10 (0.99,1.24)	
≥0	1.12 (1.02,1.24)	1.07 (0.99,1.17)	1.22 (1.05,1.43)	

Sleep duration and obesity: The joint effects model was adjusted for age, sex, education, marital status, E-DII Score, smoking status, pack year history, alcohol consumption, physical activity, hormone therapy (estrogen and progesterone), diet supplement use, family history of colon cancer, and previous chronic condition (heart disease, stroke, diabetes). Sleep duration and E-DII score: The joint effects model was adjusted for age, sex, education, marital status, BMI, smoking status, pack year history, alcohol consumption, physical activity, hormone therapy (estrogen and progesterone), diet supplement use, family history of colon cancer, and previous chronic condition (heart disease, stroke, diabetes).

## Data Availability

The data presented in this study are available upon formal request from the Multiethnic Cohort. https://www.ncbi.nlm.nih.gov/projects/gap/cgi-bin/study.cgi?study_id=phs002183.v1.p1 15/01/2025. The questionnaire at baseline can be accessed at: https://www.uhcancercenter.org/for-researchers/mec-questionnaires.

## Supplementary Materials

The following supporting information can be downloaded at: https://www.mdpi.com/article/10.3390/nu17030370/s1, Table S1: Adjusted analysis of the association between sleep duration and CRC incidence stratified by age, sex, and BMI status.

## Abbreviations

The following abbreviations are used in this manuscript

aHR	Adjusted hazard ratio
BMI	Body mass index
CIs	Confidence intervals
CRC	Colorectal cancer
E-DII	Energy-adjusted Dietary Inflammatory Index
MEC	Multiethnic cohort
