# Supplementary material for: Sleep Duration, Dietary Inflammatory Potential, and Obesity in Relation to Colorectal Cancer Incidence in the Multiethnic Cohort
Source: Nutrients. 2025 Jan 21;17(3):370. doi: 10.3390/nu17030370 (PMC11820058; doi:10.3390/nu17030370)
Supplement: Supplementary file 1 [file nutrients-17-00370-s001.zip › nutrients-3431967-supplementary.pdf]

Supplementary Table (S1):

Table S1: Adjusted analysis of association between sleep duration and CRC incidence stratified by age, sex and BMI Status

	Sleep duration		
	**Short (≤6hours) HR (95% CI)**	**Normal/adequate (7-8 hours) HR (95% CI)**	**Long (≥ 9 hours) HR (95% CI)**
**Age[1]**			
45-54	1.05 (0.92,1.18)	1.00 (Reference)	1.12 (0.90,1.41)
55-64	1.00 (0.90,1.11)	1.00 (Reference)	1.15 (0.98,1.37)
≥65	1.03 (0.94,1.13)	1.00 (Reference)	1.05 (0.92,1.21)
**Sex[2]**			
Male	1.02 (0.93,1.10)	1.00 (Reference)	1.10 (0.97,1.26)
Female	1.04 (0.95,1.13)	1.00 (Reference)	1.10 (0.95,1.27)
**BMI Status[3]**			
Underweight	1.60 (0.98,2.57)	1.00 (Reference)	0.74 (0.30,1.82)
Normal	1.02 (0.91,1.12)	1.00 (Reference)	1.08 (0.91,1.28)
Overweight	0.94 (0.86,1.04)	1.00 (Reference)	1.09 (0.94,1.27)
Obese	1.17 (1.02,1.34)	1.00 (Reference)	1.18 (0.97,1.43)
**E-DII Quartile[4]**			
Quartile 1 (−6.4 to −2.9)	0.99 (0.97,1.12)	1.00 (Reference)	1.04 (0.85,1.28)
Quartile 2 (−2.8 to −1.5)	1.09 (0.97, 1.24)	1.00 (Reference)	1.05 (0.85,1.29)
Quartile 3 (−1.4 to 0.1)	1.02 (0.90,1.16)	1.00 (Reference)	1.12 (0.92,1.36)
Quartile 4 (0.2 to 5.0)	1.02 (0.90,1.15)	1.00 (Reference)	1.15 (0.97,1.37)

[1]Adjusted for sex, education, marital status, BMI, E-DII Score, smoking status, pack year history, alcohol consumption, physical activity, hormone therapy (estrogen and progesterone), diet supplement use, family history of colon cancer and previous chronic condition (heart disease, stroke, diabetes)
[2]Adjusted for age, education, marital status, BMI, E-DII Score, smoking status, pack year history, alcohol consumption, physical activity, hormone therapy (estrogen and progesterone), diet supplement use, family history of colon cancer and previous chronic condition (heart disease, stroke, diabetes)
[3] Adjusted for age, sex, education, marital status, E-DII Score, smoking status, pack year history, alcohol consumption, physical activity, hormone therapy (estrogen and progesterone), diet supplement use, family history of colon cancer and previous chronic condition (heart disease, stroke, diabetes)
[4] Adjusted for age, sex, education, marital status, BMI, smoking status, pack year history, alcohol consumption, physical activity, hormone therapy (estrogen and progesterone), diet supplement use, family history of colon cancer and previous chronic condition (heart disease, stroke, diabetes)
